# Immunomodulatory Effects of Dopamine in Inflammatory Diseases

**DOI:** 10.3389/fimmu.2021.663102

**Published:** 2021-04-09

**Authors:** Yifei Feng, Yan Lu

**Affiliations:** Department of Dermatology, The First Affiliated Hospital, Nanjing Medical University, Nanjing, China

**Keywords:** dopamine, inflammation, inflammatory bowel disease, Parkinson disease, rheumatoid arthritis, systemic lupus erythematosus, multiple sclerosis

## Abstract

Dopamine (DA) receptor, a significant G protein-coupled receptor, is classified into two families: D1-like (D1 and D5) and D2-like (D2, D3, and D4) receptor families, with further formation of homodimers, heteromers, and receptor mosaic. Increasing evidence suggests that the immune system can be affected by the nervous system and neurotransmitters, such as dopamine. Recently, the role of the DA receptor in inflammation has been widely studied, mainly focusing on NLRP3 inflammasome, NF-κB pathway, and immune cells. This article provides a brief review of the structures, functions, and signaling pathways of DA receptors and their relationships with inflammation. With detailed descriptions of their roles in Parkinson disease, inflammatory bowel disease, rheumatoid arthritis, systemic lupus erythematosus, and multiple sclerosis, this article provides a theoretical basis for drug development targeting DA receptors in inflammatory diseases.

## Introduction

Dopamine (DA), a member of a group of neurotransmitters called “catecholamines”, relies on the conversion of tyrosine to L-DOPA by tyrosine hydroxylase (TH). Chromaffin cells in suprarenal glands and the intestine are the main sources of plasma dopamine. Other sources of dopamine are immune cells, peripheral nervous system, and central nervous system.

Dopamine receptors (DRs) are mainly divided into D1-like (D1 and D5) and D2-like (D2, D3, and D4) receptors, which display different affinities for dopamine: D3R>D5R>D4R>D2R>D1R(Ki(nM) = 27, 228, 450, 1705, 2340, respectively) (1). As members of monoaminergic G protein-coupled receptor (GPCR) family, they not only regulate behavior, movement, and endocrine but are also important molecules connecting the nervous system and immune system. Integrating the knowledge acquired by those studies, evidences indicate that stimulation of low-affinity DRs are coupled to anti-inflammatory mechanisms, while high-affinity DRs have opposite effects (**Table 1**).

**Table 1:** Dopamine receptor-mediated signaling pathways in inflammatory diseases.

Diseases	Locations	DRs		Pathways	Animal experiments	Clinical trials
Parkinson Disease	Astrocytes and microglia	D1R		AC/cAMP/NLRP3/caspase-1	(2)	–
				cAMP/PKA/NF-κB/M1 phenotype	–	–
				cAMP-IL-4/C/EBP/arginase-1/Fizz1	–	–
		D2R	D2R	CRYAB/STAT3/NF-κB	(3)	–
				β-arrestin2/NLRP3	(4)	–
				macrophage phagocytic activity	(5)	–
				AT1/NADPH-oxidase/superoxide axis	(6)	–
			A2A-D2R heteromers	cross-antagonistic effects	(7)	–
				striatal glutamatergic transmission		–
		D3R		Fizz1	(8, 9)	–
				Th1, Th17 differentiation/IFN-γ, TNF-α/M1 phenotype	(9)	–
	Striatum	D3R		DAT-MAO-VMAT2/DA concentration	(10)	(11, 12)
				α-Syn/fibril formation	(13, 14)	
				LC3-Beclin1/autophagy-dependent degradation of toxic fibrils	(15)	
				bi-directional regulation with BDNF	(16)	
				GSH and GSH peroxidase/ROS	(17)	
				neurogenesis in the nigrostriatal pathway	(18, 19)	
		Heteromers	D1-D3R heteromers	D1R/Shp-2/Erk1/2	(20–22)	(23)
				a switch from G protein-dependent to G protein-independent D1R-mediated signaling		
			A2A-D2R heteromers	cross-antagonistic effects	(24)	(24–26)
				Striatopallidal GABA/enkephalin pathway		
			A1-D1R heteromers	cross-antagonistic effects	(27)	–
				GABAergic transmission from striato-nigral terminals		–
			H3-D1R heteromers	cross-antagonistic effects	(28–30)	–
			NMDA-DR heteromers	mutual promotion effects in NMDA-D1R heteromers	(31)	–
				cross-antagonistic effects in NMDA-D2R heteromers		–
			A2A-mGlu5-D2R heteromers	cross-antagonistic effects	(29)	–
				mGlu5 desensitization		–
			A2A-CB1-D2R RM	cross-antagonistic effects	(32)	–
	Hippocampus	D1R		Wnt/β-catenin signaling	(33)	–
		D2R		TCF/LEF site/Wnt3a/cell proliferation	(33)	–
				Wnt/β-catenin signaling		–
Inflammatory bowel disease	T cells	D3R		Tregs/IL-10	(34)	–
				gut-tropism		–
				Th1 and Th17 differentiation		–
	Dendritic cells	D5R		ROR-γt, IL-12/23/Th1, Th17 differentiation	–	–
		D2R		IL-10	(35)	–
				VEGF–VEGFR2		–
Rheumatoid Arthritis	CD4+T cells	D1R		Th17/Treg balance	(36)	–
		D2R			(37)	–
	Mast cells	D3R		TLR4/MAPK/TNF signaling	(38)	–
				TLR4/NF-κB/TNF signaling		–
	B cells	D2R		TNF-α	–	–
	Fibroblasts	D1R		release of IL-6 and IL-8	–	(39)
				cell migration	–	(40)
	Osteoclasts	D2R		cAMP/PKA/CREB/RANKL	(41)	–
Systemic lupus erythematosus		D2R		Tregs/CD4+T cells	–	–
				β-arrestin-glycogen synthase kinase-3-dependent pathway/PRL	(42)	(43)
		D4R		T cell proliferation	–	–
Multiple sclerosis	Dendritic cells	D5R		IL-23/Th17 differentiation/IL-17	(44, 45)	–
				IL-12/Th1/IFN-γ	–	–
	Tregs	D5R		Tregs/Teffs	–	(46)

-, no supporting experiments; DRs, dopamine receptors; AC, adenylyl cyclase; cAMP, cyclic AMP; NLRP, Nod-like receptor protein; PKA, protein kinase A; C/EBP, CCAAT/enhancer-binding protein; CRYAB, α-B crystallin; STAT, signal transducer and activator of transcription; AT1, angiotensin II type 1; NADPH, nicotinamide adenine dinucleotide phosphate; TNF, tumor necrosis factor; DAT, dopamine transporter; VMAT, vesicular monoamine transporter; DA, dopamine; BDNF, brain-derived neurotrophic factor; GSH, glutathione; ROS, reactive oxygen species; ERK, extracellular signal-regulated kinase; GABA, γ–aminobutyric acid; A2AR, adenosine A2A receptor; H3R, histamine H3 receptor; mGlu5, metabotropic glutamate type 5; CB, cannabinoid; RM, receptor mosaic; VEGF, vascular endothelial-derived growth factor; TLR, Toll-like receptors; MAPK, Mitogen-activated protein kinase; CREB, cAMP-response element binding protein; RANKL, receptor activator of nuclear factor-κB ligand; PRL, prolactin.

## Structure

DRs and other GPCRs can form homodimers as well as heteromers with receptors from other superfamilies.

### Homodimers

It is a general physical property of the class-A GPCRs to form transient homodimerization (47), which can be confirmed by the minimal single functional unit consist of D2R homodimers and a G protein, mediated by direct association among receptors (48).

### Heteromers

#### DR-DR Heteromers

##### D1-D2R Heteromers

Activation of the Gq-coupled D1-D2R heteromers in striatal neurons results in PLC-dependent intracellular calcium release, thus activating calmodulin-dependent protein kinase II (CaMKII)/Mutations of the methyl-CpG binding protein 2 (MeCP2)/brain-derived neurotrophic factor (BDNF) pathway (49).

##### D1-D3R Heteromers

D1-D3R heteromers, located in the ventral striatum, enhance D1R agonist affinity, the potency of D1R in activating adenylyl cyclase (AC), impair agonist-induced D1R internalization (20), and induce a switch from G protein-dependent to G protein-independent D1R-mediated signaling, including MAPK and AKT (50).

##### D2-D4R Heteromers

D2R can exist in a heterodimeric form with D4R, participating in dopamine-induced decrease of K^+^-induced glutamate release (51).

#### Adenosine Receptor-DR Heteromers

##### A2A-D2R Heteromers

The C-terminal tail of the A2AR and the intracellular loop3 of the D2R form antagonistic heteromers through direct electrostatic interactions in striatopallidal GABAergic neurons and glial cells (52), resulting in a rapid switch from D2R-Gi coupling toward β-arrestin2/Akt/GSK-3 signaling (53, 54), and reducing the activity of the striatopallidal GABA/enkephalin-mediated inhibition of the excitatory glutamate thalamocortical pathway through Ca2+ and cAMP/PKA pathway whose activation involves the phosphorylation of different PKA substrates, including DARPP-32, CREB, and α-amino-3-hydroxy-5-methyl-4-isoxazole-propionic acid receptor (AMPAR) (55, 56). In addition, A2AR agonists and antagonists produce the same allosteric modulation of D2R agonist binding when individually administered, the effect of which is offset when co-administered (57).

##### A2A-D3R Heteromers

A2AR activation reduces D3R agonist affinity and the ability of D3R to inhibit AC (58).

##### A1-D1R Heteromers

There exist antagonistic intramembrane A1-D1R interactions at the AC level in the dorsal, ventral striatum, and prefrontal cortex (59). A1R activation in the A1–D1R heteromers leads to an uncoupling of the D1 receptor to its Gs/olf protein, modulates D1R antagonist binding sites that cause a reduction of their affinity, and offsets D1R-induced GluA1 phosphorylation that facilitates AMPA glutamate transmission (60). In addition, A1R signaling inhibits excitatory synaptic transmission through the inhibition of glutamate release, ionotropic glutamate receptors, and neuronal excitability *via* inward rectifying, cAMP/PKA/DARPP-32 pathway, as well as calcium-activated potassium channels (61, 62).

#### H3-DR Heteromers

H3-D1R and H3-D2R heteromers, in which H3R potentiates the D2R-induced inhibition of the indirect pathway and inhibits the D1R-induced excitation of the direct pathway in AC level, modulate DA and GABA release (63) and subsequent locomotor activation. H3-D1R heteromers also couple to Gi to direct histaminergic input towards β-arrestin/MAPK pathway within the GABAergic neurons (64) with an increased phosphorylation of rpS6 and transient phosphorylation of GSK3β (65), and affect both rapid receptors signaling like Ca2+ mobilization and longer cell signaling pathways like p38, involving neuronal cell death (66). While H3-D2R heteromers regulate Akt-GSK3β, producing a more slowly developing dephosphorylation (65).

#### *N*-methyl-D-aspartate (NMDA)-DR heteromers

##### NMDA-D1R Heteromers

Heteromers, consist of D1R and NMDAR, increase the phosphorylation of NR1 and NR2B subunits, surface insertion of NR2B, and NMDA-triggered Ca2+ upregulation *via* cAMP/PKA/RACK1-mediated Fyn activation (67, 68), and PKC/CAKβ/Src signaling (69), thus enhancing NMDAR-mediated currents. While D1R, coupled to NR2A subunit, inhibits NMDA receptor-gated currents through a reduction in cell surface receptor numbers (70, 71). In addition, NMDA-D1R heteromers participate in the attenuation of NMDA receptor-mediated excitotoxicity *via* PI-3K/Akt/GSK3β pathway instead of modulating Ca2+ influx (70, 72). Besides, D1R-mediated cAMP/PKA/DARPP-32 signaling, Ca2+ pathway, and tyrosine phosphorylation of NR2B subunit allow for the activation of NMDA-mediated ERK, a signal integrator for dopamine and glutamate neurotransmission (73).

NMDAR abolishes D1R internalization and enhances D1R-mediated cAMP accumulation *via* a SNARE-dependent mechanism (74, 75).

##### NMDA-D2R Heteromers

In glutamate synapses, NMDA-D2R heteromers, in which ICL3 of D2R interacts with the NR2B subunit, interfere with the binding of Ca2+/CaMKII to NR2B, reduce NR2B phosphorylation, and inhibit NMDA receptor-mediated currents (76).

#### Other Heteromers

##### Cannabinoid Type 1(CB1)-DR Heteromers

CB1-DR heteromers in the striatum (32) modulate D1R and D2R function in an opposite fashion. Stimulation of either CB1 or D2R results in Gαi signaling, while simultaneous co-activation of both receptors switches coupling from Gαi to Gαs proteins (77).

##### D3R-nAChR Heteromers

D3R-nAChR heteromers in DA neurons are the molecular unit involved in the induction of neurotrophic effects, neuroprotection, and inhibition of α-syn accumulation (78).

### Receptor Mosaic (RM)

#### A2A-mGlu5-D2 RM

A2AR and mGlu5 act synergistically to counteract the D2R signaling in striatopallidal neurons, reducing mGlu5 desensitization (79), and exciting the striatopallidal GABA neurons with firing and altered gene expression (80, 81).

#### A2A-CB1-D2 RM

A2A-CB1-D2 RM in striatopallidal neurons selectively couples to the mitogen-activated protein kinase pathway (82). The binding of A2AR and CB1R agonists decreases D2R agonist affinity (32).

## Roles of Dopamine in Inflammation

### NLRP3 Inflammasome

**Figure 1:**
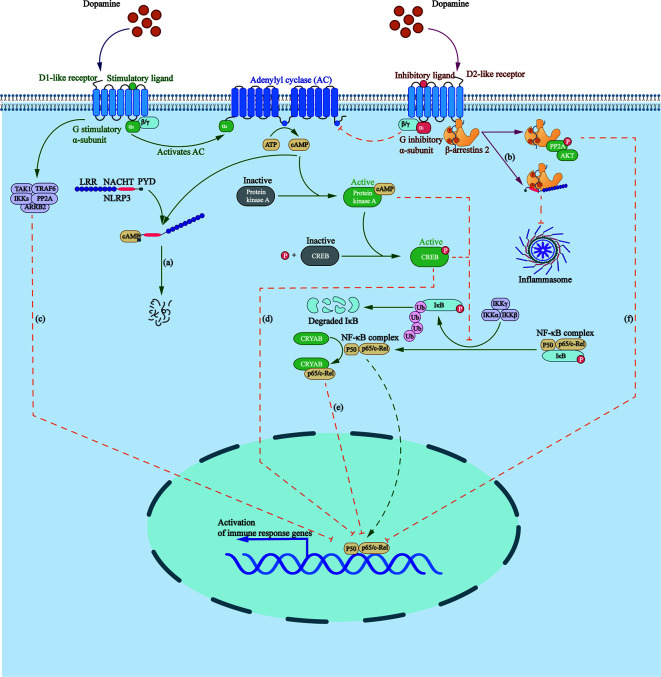
Roles that dopamine receptors play in regulating inflammasome formation as well as NF-κB pathway. **(A)** Elevated cAMP, induced by D1 like receptors, directly binds to NLRP3, triggering the ubiquitination of NLRP3 NACHT and LRR domains with K48 ubiquitin chains by MARCH7, targeting NLRP3 to autophagy-mediated degradation. **(B)** β-arrestin2 recruited by D2R binds to NLRP3 to repress its activation. **(C)** D5R directly recruits TRAF6, ARRB2, TAK1, IKKs, and PP2A to form a multiprotein complex, impairing TRAF6-mediated activation of NF-κB. **(D)** Activated cAMP/PKA/CREB signaling inhibits p65/RelA and p50 activation as well as their DNA binding by delaying IKB-α degradation and competing for the KIX binding site on CREB binding protein. **(E)** D2R signaling increases the level of CRYAB, which combines with NF-κB p65, thus negatively regulating the NF-κB signaling pathway. **(F)** D2R activation increases the expression of PPP2R2C, leading to PP2A and Akt dephosphorylation, and to the inhibition of the IKKα/IκBα/NF-κB signaling pathway.

NLRP3 inflammasome is a group of intracellular multi-protein complexes, participating in the pathogenesis of a variety of diseases, including inflammatory bowel disease, gout, atherosclerosis, and Alzheimer’s disease (83, 84). It was found that DA can inhibit lipopolysaccharide (LPS)-induced activation of NLRP3 inflammasome and subsequent production of caspase-1 and IL-1β in a time- and dose-dependent manner through G protein pathway and β-arrestin–dependent pathway (**Figure 1**).

D1-like DRs are coupled to the stimulatory G-subunit, Gαs, whereas D2-like DRs, are coupled to the inhibitory G-subunit, Gαi. D1-like DRs stimulate the activity of AC by activating Gαs/olf, thus promoting the production of cAMP, which directly binds to NLRP3, triggering the ubiquitination of NLRP3 NACHT and LRR domains with K48 ubiquitin chains by the E3 ubiquitin ligase membrane associated ring-CH-type finger 7, targeting NLRP3 to autophagy-mediated degradation (2, 85).

β-arrestin is involved in the negative feedback regulation of inflammatory process in sepsis, asthma, rheumatoid arthritis, and other inflammatory diseases (86). β-arrestin2 recruited by D2R functions as the downstream protein of GPR120 and GPR40 to repress inflammasome activation *via* binding to NLRP3 in a concentration-dependent manner (87, 88).

### NF-κB Signaling

An inactive form of NF-κB consists of a three-subunit complex: two DNA-binding subunits of p50 and p65/RelA, and an inhibitory subunit called IκB (**Figure 1**). Activation of NF-κB requires the phosphorylation and degradation of IκBα by ubiquitin–proteosome pathway, contributing to translocation of NF-κB dimer into the nucleus and the transcription of inflammatory genes, including cyclooxygenase-2, inducible nitric oxide synthase, tumor necrosis factor-α (TNF-α), and interleukin-6 (IL-6) (89).

D1R participates in the regulation of inflammation through cAMP/PKA/NF-κB pathway. Elevated cAMP activates PKA and phosphorylates cAMP-response element binding protein (CREB), inhibiting p65/RelA and p50 activation as well as their DNA binding ability by delaying IKB-α degradation and competing for the KIX binding site on CREB binding protein (90).

D5R inhibits NF-κB signaling by mediating the negative regulation of ARRB2/PP2A on TRAF6-dependent signaling. A study found that D5R, *via* the EFD and IYX(X)I/L motifs in its CT and IC3 loop, respectively, can directly recruit TRAF6 and its negative regulator ARRB2, as well as downstream signaling proteins, such as TAK1, IKKs, and PP2A, to form a multiprotein complex, which impairs TRAF6-mediated activation of NF-κB (91).

D2R negatively regulates the NF-κB pathway. First, the pathway downstream D2R activation leads, *via* increasing the expression of PPP2R2C, to PP2A and Akt dephosphorylation, and the inhibition of the IKKα/IκBα/NF-κB pathway as well as the expression of NLRP3 mRNA (2, 92). Second, D2R signaling increases the level of αB-crystallin, also called CRYAB, which is known as a small heat-shock protein with neuroprotective and anti-inflammatory activities (93). D2R agonists were found to promote a direct combination between CRYAB and NF-κB p65 (92) and enhance the interaction between CRYAB and STAT3, blocking its DNA binding activity (94).

### Immune Cells

#### T Cells

Dopamine dynamically regulates the immune response of T cells through DRs, depending on the concentrations of dopamine, the activation states of T cells, and the types and subtypes of T cells. Dopamine concentrations can be divided into three gradients: 10 nM, 1 μM, and 0.1 to 1 mM. Dopamine’s optimal concentration for inducing a physiological and specific effect on resting T cells turns out to be low: 10 nM, in which dopamine activates normal resting/primeval effector cells or improves the continuous important cell function, and inhibits activated T cells. Dopamine at a concentration range of 0.1 to 10 μM still affects T cells, but the potency and specificity are lower. At a very high concentration of 0.1 to 1 mM, dopamine’s effect is non-specific and even toxic (95).

Tregs are inhibitory T cells, mainly inhibiting the activity of Teffs. D1-like DRs on the surface of Tregs reduce their inhibitory activity, as well as the production of IL-10 and TGF–β (96), the effects of which are significantly attenuated in activated Tregs (97). Instead, activation of D1-like DRs in Teffs does not lead to self-inhibition.

According to cytokines produced during T cell activation, naive CD4+ T cells undergo differentiation into specific effector phenotypes, including Th1, Th2, and Th17. IFN-γ and IL-12 induce Th1 differentiation, while IL-4 is the primary inducer of differentiation into Th2, and IL-6 and TGF-β together induce Th17 phenotype (98). In activated CD4+ T cells, D3R signaling, preferentially activated at lower dopamine concentrations, enhances the production of IFN-γ (99) and reduces the synthesis of IL-4 and IL-10 and the expression of SOCS5 (100), a side-regulator of Th2 differentiation. Besides, D3R stimulating reduces cAMP level and extracellular signal-regulated kinase 1/2 (ERK1/2) phosphorylation, resulting in enhanced activation of CD4+ T cells and Th1 differentiation (101).

#### Dendritic Cells

Dendritic cells (DCs) have been shown to synthesize and store dopamine, which is released to the original CD4 + T cells during DC-T cell interaction, thus affecting the differentiation of CD4+ T cells. The expression of D1R and D5R is more than that of D2R and D3R on the DC surface. D1-like DRs-mediated increase in cAMP promotes TH phosphorylation, thus boosting the synthesis of dopamine and Th2 differentiation, while D2-like DRs play an opposite role (95).

However, D5R and D2R have different effects. D5R signaling significantly enhances the production of LPS-induced IL-23 and IL-12 (44), inducing Th1/Th17 differentiation and the activity of B cell-activating transcription factor, increasing the expression of Th17 transcription factors, like ROR-γt (102). Besides, stimulation of D2R induces a significant human monocyte-derived DC-mediated Th2 differentiation and suppresses the secretion of inflammatory cytokines (103).

#### Monocytes and Macrophages

**Figure 2:**
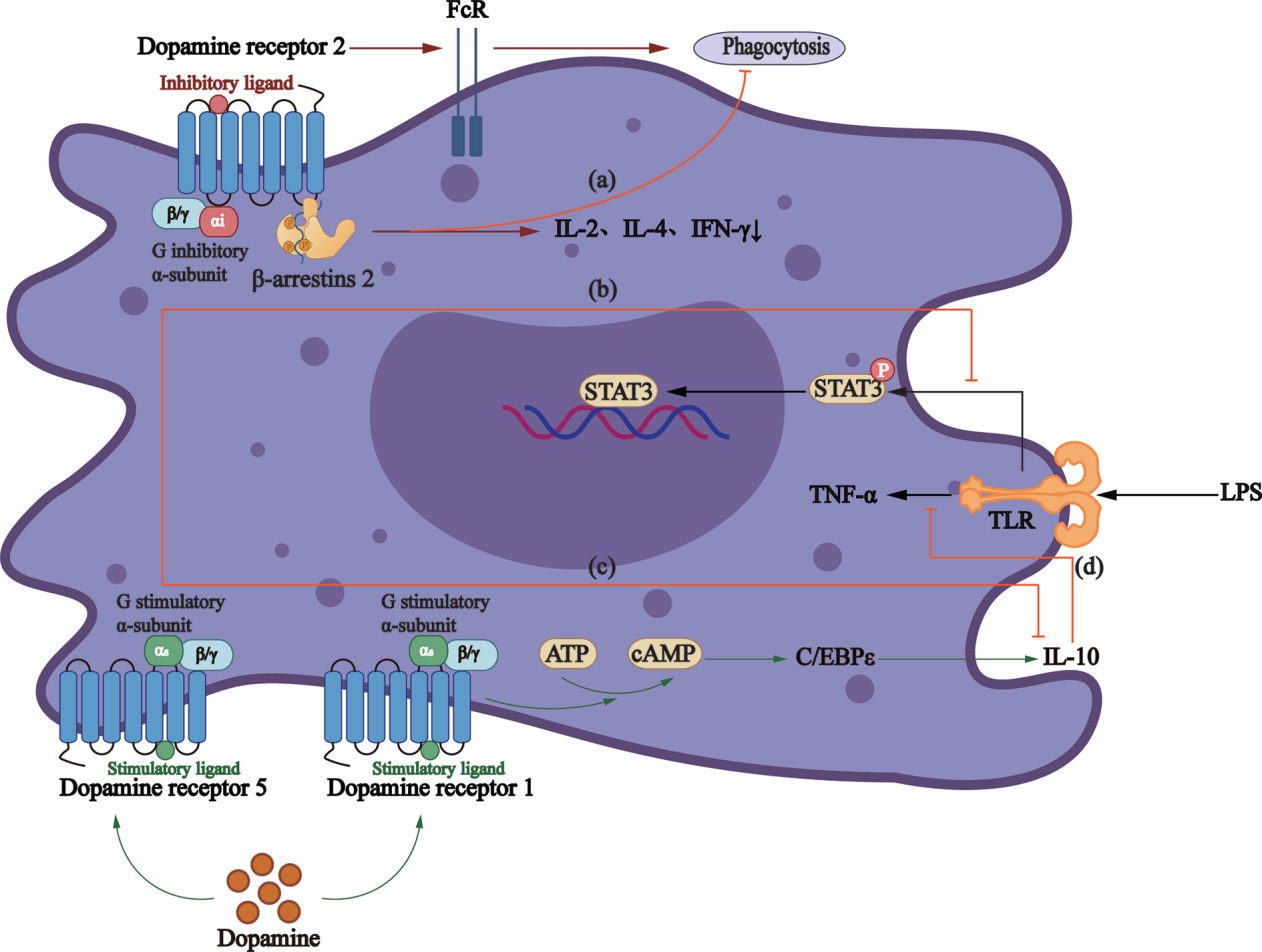
Roles that dopamine receptors play in macrophages. **(A)**The activation of D2-like DRs can regulate the phagocytic activity of macrophages through β-arrestin2 pathway, and reduce the secretion of IL-2, IL-4, and IFN-γ. **(B)** LPS increases TNF - α production *via* TLRs and mediates activation of STAT3, which can be inhibited by D5R signaling. **(C)** Dopamine reduces the production of anti-inflammatory factor IL-10 through D5R. **(D)** D1R/cAMP/C/EBPϵ signaling increases IL-10 production, thus inhibiting LPS-mediated production of TNF-α.

D2-like DRs participate in a cAMP-independent pathway to regulate macrophage phagocytic function, thus reducing the secretion of IL-2, IL-4, and IFN-γ (104), the effect of which is concentration-dependent. Studies demonstrated that D2-like DRs decrease the production of TNF-α, IL-6, and CCL2 at 10^−8^ M concentration (105), while increase the production of IL-6 significantly at 2 and 20 × 10^−6^ M (106) concentration. Conversely, Gomez et al. proposed that another mechanism is to increase the expression of the Fcγ receptor, thus increasing the phagocytosis of macrophages (107) (**Figure 2**).

A study showed that D1R signaling blocks the function of LPS-activated macrophages and monocytes and production of inflammatory cytokines (108). Also, the elevated cAMP can indirectly activate CCAAT/enhancer-binding protein, together with which CREB/activating transcription factors are believed to be the major cause of IL-10 production by monocytes (109). However, D5R in monocytes obtained from MS patients involves the reduction of STAT3 activation, a transcription factor that limits the production of IL-12 and IL-23 (110). A study in HIV patients demonstrated that dopamine promotes the inflammatory phenotype of macrophages through D5R at physiological concentration and reduces the production of IL-10 (105).

## Inflammatory Diseases

### Parkinson Disease

Parkinson disease (PD) is the second most prevalent central nervous system degenerative disease, characterized by slow and progressive loss of midbrain substantia nigra dopamine with the accumulation of α-synuclein in Lewy bodies and neuritis (111).

#### Astrocytes and Microglia

DRs expressed on astrocytes and microglia have been confirmed to participate in the pathogenesis of chronic nervous system inflammatory diseases. Increases in D1R and D4R, and decreases in D3R, D5R mRNA expression are showed in a study analyzing the mRNA expression of all five DRs in BV2 microglial cells in response to LPS (112). It is noteworthy that these anti-inflammatory effects exerted by dopaminergic signaling in astrocytes are mediated by D1R and D2R, while D3R mediates the pro-inflammatory effects (8).

##### D1R

D1R signaling participates in the negative regulation of the activation of NLRP3 inflammasome, which can be assembled upon stimulation with accumulated endogenous metabolites such as fibrillar amyloid β and 25-hydroxycholesterol (113), the subsequent secretion of caspase-1 and IL-1β contributes to the destruction of dopaminergic neurons (114). A study reported that caspase-1 can process α-synuclein into a truncated, aggregation-prone form that facilitates its aggregation (115), thus participating in activation of NF-κB and expression of Toll-like receptors (TLRs).

Microglia can be divided into M1 type and M2 type phenotypes. M2 microglia promotes the release of anti-inflammatory factors, such as TGF-β and IL-10, while M1 microglia plays an opposite role (116). Studies have shown that D1R signaling inhibits the activation of M1 phenotype by cAMP/PKA/NF-κB pathway. Besides, cAMP, along with IL-4, activates CCAAT/enhancer-binding protein (C/EBP) to induce the expression of M2 regulatory genes (such as arginase-1), thus reducing the expression of M1-related inflammatory factors and increasing the expression of M2-related anti-inflammatory mediators (such as FIZZ1) (117).

##### D2R

Several lines of evidences suggest that D2R signaling alleviates neuroinflammatory injury by CRYAB/STAT3 pathway, β-arrestin2/NLRP3 pathway, and its regulation of macrophage phagocytic activity (4, 5, 94, 118). A2AR inhibition of D2R signaling regulates striatal glutamatergic transmission dysfunction *via* increasing the extracellular glutamate levels (119) and promotes microglia-mediated neuroinflammation (120). Besides, D2R modulates astroglial and microglial activity *via* decreasing the microglial AT1/AT2 ratio, thus inhibiting AT1/NADPH-oxidase/superoxide axis, based on AT1-D2R heteromers (6).

##### D3R

Genetic deficiency of D3R, attenuated neuroinflammation and subsequent neurodegeneration on a murine model of PD induced by acute intoxication with 1-methyl-4-phenyl-1,2,3,6-tetrahydropyridine (121), related to the limited basal production of Fizz1 (8) and the acquisition of M1 phenotype. Besides, the high levels of IFN-γ and TNF-α, secreted by D3R signaling-induced Th1 and Th17 differentiation, lead to M1 phenotype (122), confirmed by a study that compared with the control group, PD patients have increased Th1 cells and Th17 cells but decreased Tregs (123).

However, experimental results showed decreased D3R expression on CD4+ T cells in the peripheral circulation of PD patients, which might be due to a compensatory mechanism attempting to reduce the inflammatory effect (124, 125). Another plausible explanation was that CD4+ T cells with high D3R expression were specific for α-synuclein, thus these cells could only be detected at the site of inflammatory infiltration, instead of the peripheral circulation (124).

#### Striatum

##### D3R

D3R expressed on striatal neurons can raise dopamine concentration, decrease α-Syn accumulation, enhance secretion of BDNF, ameliorate neuroinflammation, alleviate oxidative stress, and promote neurogenesis in the nigrostriatal pathway.

D3R increases the content of DA in the synaptic cleft by impeding DAT’s reuptake of DA, inhibiting MAO to reduce DA decomposition, and promoting the release of DA by VMAT2. Further, D3R activation hinders the phosphorylation of α-Syn to inhibit fibril formation (126). Besides, D3R activation enhances autophagy-dependent degradation of toxic fibrils by modulating autophagy constituent proteins LC3 and autophagy-related protein Beclin1 (15). It was found that D1, D2, and possible D1-D2 receptor heteromers can activate BDNF receptors in striatal neurons (127). Also, D3R can jointly protect striatal neurons through its bi-directional regulation with BDNF, a high level of which may ameliorate symptoms in PD patients (128). Experiments have shown that D3R agonists can normalize glutathione (GSH) and GSH peroxidase levels in animal models of PD (17), thereby reducing ROS-induced damage, however, some studies showed no association.

##### Heteromers

Evidences for the existence of DR heteromers in the striatum (see *Structure*) provide novel targets in treating PD and other brain disorders. This section is a supplement to clinical implications of heteromers on treatment response and prognosis of PD.

Striatal D1-D3R heteromers are closely correlated with age of onset, PD stage, dopamine responsiveness, and survival time (129). Besides, the expression of D3R induced by long-term L-DOPA treatment aggravates D1R oversensitivity and is correlated with the severity of LID, *via* activating D1R/Shp-2/Erk1/2 pathway (130).

Chronic administration of the histamine H3 receptor agonist immepip decreases L-Dopa-induced dyskinesias (28), while a combination of D2 agonists and inhibitors of endocannabinoid degradation improves parkinsonian motor deficits (131).

NMDAR antagonist MK-801 aggravates D1R-induced dyskinesias, while effectively reduces D2R-induced dyskinesias, the degree of which is of the same magnitude as the reduction of L-DOPA-induced dyskinesias (31).

There is an increase in the therapeutic index and locomotor improvement of L-DOPA with adenosine A2AR antagonists, like istradefylline (25, 132) and tozadenant (26), and/or D2R agonists, based on the existence of A2A–D2R heteromers (24, 133), which function also as a biomarker to monitor PD (134). Besides, adenosine A1 receptor stimulation reduces D1 receptor-mediated GABAergic transmission from striato-nigral terminals and attenuates L-DOPA-induced dyskinesia in dopamine-denervated mice (27).

In addition, simultaneous blockade of both mGlu5 and A2AR in A2A–mGlu5-D2R RM increases their efficacy in reversing parkinsonian deficits (29). While a study showed that A2A-CB1-D2 RM expression attenuates in L-Dopa-treated PD monkeys with abolished negative cross-talk (32).

#### Hippocampus

DRs are widely expressed in the hippocampus. D1R can upregulate Wnt/β-catenin signaling in the hippocampus of PD rats, leading to the enhanced NSC proliferation, long-term survival, and neuronal differentiation (33). Besides, D2R-dependent cross-talk modulates Wnt3a expression *via* Wnt/β-catenin signaling and an evolutionarily conserved TCF/LEF site within the WNT3A promoter, thus modulating cell proliferation (135).

### Inflammatory Bowel Disease

Inflammatory bowel disease (IBD) is a group of chronic gastrointestinal inflammatory diseases including Crohn’s disease (CD) and ulcerative colitis (UC). Dopamine in IBD can be produced from enteric nervous system, the intestinal epithelial layer, and certain immune cells. Interestingly, inflamed mucus from IBD patients show a significant reduction of dopamine, mainly related to reduced dopamine uptake and the number of sympathetic fibers interacting with the intestinal wall (136).

Reduced intestinal dopamine levels [≈140 pg/ml in healthy individuals;≈45 pg/ml in CD and UC patients (137)] play a pro-inflammatory role by activating D3R and D5R. On one hand, D3R signaling depresses the immunosuppressive potency of Tregs, attenuates IL-10 production, and limits the acquisition of gut-tropism (34). On the other hand, Th1/Th17 differentiation, induced by increased D3R expression on intestinal CD4+ T cells, the induction of ROR-γt expression by D5R signaling in dendritic cells, and the increased IL-23 and IL-12 resulting from D5R signaling, contributes to the persistence of chronic inflammation (138).

Conversely, high concentrations of dopamine in the intestine of healthy people can stimulate D2R, promoting the production of IL-10, inhibiting intestinal motility and ulcer development (139), as well as playing a role of the negative regulator of VEGF–VEGFR2-mediated increase in vascular permeability (35, 140, 141), thus controlling the development of IBD.

### Rheumatoid Arthritis

Rheumatoid arthritis (RA) is a systemic inflammatory autoimmune disease characterized by persistent inflammation of the joint synovium (142). Dysregulated immune signals, such as dopamine, control bone remodeling *via* affecting osteoclasts differentiation or the secretion of pro-inflammatory cytokines.

#### CD4+ T Cells

Dopamine released by DCs contributes to the Th17/Treg imbalance *via* the IL-6-Th17 axis and causes aggravation of synovial inflammation. A study showed that D2-like DRs agonist improves Th17/Treg imbalance by downregulating the expression of Th17-related pro-inflammatory cytokines but upregulating Treg-related anti-inflammatory cytokine expression (143), the effect of which can be suppressed by selective D2-like DRs antagonist.

#### Mast Cells

D3R on bone marrow-derived mast cells may negatively regulate LPS-induced TLR4 expression and its downstream production of TNF and other cytokines (38), thus effectively inhibiting the production of ROS and reducing joint inflammation in RA patients. With the increase in RA severity degree, D3R-positive MCs in the synovial fluid are gradually reduced, led by ROS production, reduced antioxidant capacity, reduced cell membrane stability, and increased sensitivity of membrane components to a damaging agent, which are negatively correlated with the level of MDA and protein carbonylation (144).

#### B Cells

B cells have unique bone action properties. D2R expression on B cells in RA patients is negatively correlated with disease activity (145), concerning the descending TNF-α level.

#### Fibroblasts

Synovial fibroblasts (SF) are resident cells of the intimal lining layer of synovial tissue. SFs have an intact endogenous dopamine system in which D1R is overexpressed, promoting the migration of RASF cells, leading to a strong increase of SF migration in young patients (146), and decreased release of IL-6 and IL-8. However, some experiments demonstrated that the inhibitory effect on IL-8 release is not significant (146). These findings suggest that DRs expressed on synovial fibroblasts in RA patients may mainly participate in cell migration rather than inflammatory processes.

#### Osteoclasts

Osteoclasts are tissue-specific macrophage polykaryons that arise from the differentiation of monocyte/macrophage precursor cells at or near the bone surface, whose maturation and activation are mainly related to the activation of the RANK signaling (147). It was found that dopamine significantly inhibits the formation of osteoclast in a dose-dependent manner, mainly related to the restraint of RANKL-mediated expression of c-Fos and NFATc1 in the preosteoclast by D2R-induced cAMP/PKA/CREB pathway (41).

### Systemic Lupus Erythematosus

Systemic lupus erythematosus (SLE) is an autoimmune disease characterized by the involvement of kidneys and brain, with under-expressed D2R and overexpressed D4R on peripheral blood mononuclear cells (PBMCs) (148).

D2R promotes the activation and differentiation of CD4+T cells by regulating the polarization of Treg (149). D2R agonist, such as bromocriptine, suppresses PRL secretion to decrease HPRL and to normalize the dopaminergic system in SLE, through the pertussis toxin (PTX)-sensitive Gi/o and PTX-insensitive Gz proteins, as well as a G protein-independent, β-arrestin/glycogen synthase kinase-3-dependent pathway (150, 151).

Studies have shown that the stimulation of D4R on human T cells promotes quiescence (152), and overexpression of D4R in SLE patients may act as a compensatory mechanism to inhibit uncontrolled T cell proliferation, an important link in the pathogenesis of SLE.

### Multiple Sclerosis

Multiple sclerosis (MS) is an inflammatory autoimmune disorder of central nervous system (CNS), correlated with Tregs dysfunction, enhanced Th1 and Th17 responses, and autoreactive B cell overactivity.

#### DCs

There is a decreased expression of D5R in PBMCs in untreated MS patients (153), and an increase in patients treated with IFN-β. Th cell subsets involved in the pathogenesis of MS include Th1 and Th17 lymphocytes (154). D5R expressed on DCs plays a role in MS by regulating Th1 and Th17 differentiation, γδT cell functions, and GM-CSF-producing CD4+T cells *via* STAT3/NF-κB/IL-6/12/23 pathway, and is correlated with disease severity (155).

#### Tregs

D5R, which functions as a negative immunomodulator of TH and Tregs’ inhibitory activity, is up-regulated in Tregs from untreated MS patients, resulting in neuronal damage and neuroinflammation (46). Besides, D3R expression in Tregs is unaltered in untreated MS patients but significantly decreases after IFN-β treatment. A recent study showed that increased D3R and D5R mRNA expression in Tregs may be associated with the risk of MS at twelve months (156).

## Conclusions

Dopamine receptor, a significant G protein-coupled receptor, is classified into two families: D1-like DRs and D2-like DRs, with further formation of homodimers, heteromers, and receptor mosaic. Dysfunction of the systemic or local dopaminergic system during inflammation has been found in animal models and patients with various inflammatory diseases, such as Parkinson disease, inflammatory bowel disease, rheumatoid arthritis, systemic lupus erythematosus, and multiple sclerosis, indicating an important role that DRs play in inflammatory diseases. As described in this review, DRs regulate the release of inflammatory mediators and subsequent pathological processes by interacting with inflammasomes, inflammatory pathways, and immune cells, depending on different immune cells, receptor subtypes, and disease models. In conclusion, a comprehensive understanding of the relationship between DRs and inflammation will provide new insights into the inhibition of inflammatory responses by targeting dopamine receptors and ultimately contribute to the development of drugs to treat inflammatory diseases.

## Author Contributions

YFF and YL contributed to the conceptual design, writing, editing, and generation of figures for this manuscript. All authors contributed to the article and approved the submitted version.

## Funding

The authors acknowledge the financial support from Nanjing Medical University (Project approval No.81872541).

## Conflict of Interest

The authors declare that the research was conducted in the absence of any commercial or financial relationships that could be construed as a potential conflict of interest.
